# The role of immunoproteasome in diabetes and diabetes-related complications

**DOI:** 10.1016/j.gendis.2025.101861

**Published:** 2025-09-20

**Authors:** Mengwen Wang, Lingyun Luo, Lei Dai, Hesong Zeng, Hongjie Wang

**Affiliations:** a Division of Cardiology, Department of Internal Medicine, Tongji Hospital, Tongji Medical College, Huazhong University of Science and Technology, Wuhan, Hubei 430030, China; b Hubei Provincial Engineering Research Center of Vascular Interventional Therapy, Wuhan, Hubei 430030, China; c Department of Cardiology, Tongji Xianning Hospital, Xianning, Hubei 437011, China

**Keywords:** Diabetes, Diabetes-related complication, Fibrosis, Immunoproteasome, Inflammation, ONX0914

## Abstract

The immunoproteasome represents a specialized isoform of the proteasome that is integral to the processes of antigen presentation and protein degradation. While it is primarily expressed in hematopoietic cells, its expression can also be induced in non-hematopoietic cells in response to various inflammatory stimuli. Recent research has highlighted the role of the immunoproteasome in modulating islet β-cell apoptosis and glycolipid metabolism, both of which are critical mechanisms in the pathogenesis of diabetes. Furthermore, the immunoproteasome has been demonstrated to play a significant role in the development of diabetic complications through the activation of various downstream cytokines. Investigating how the immunoproteasome is activated and involved in the pathophysiological processes of diabetes and its complications may provide innovative and promising approaches for diabetes treatment. This review aims to present a comprehensive summary of current research on the role of immunoproteasome in diabetes and its associated complications, ultimately identifying novel strategies for diabetes management and therapy.

## Introduction

The immunoproteasome, identified in 1994, differs from the standard proteasome due to its function in class I antigen presentation.^1^ The proteasome is part of the ubiquitin-proteasome system, responsible for approximately 80% of protein degradation in eukaryotic cells.^2^^,^^3^ The immunoproteasome, a variant of the proteasome found primarily in hematopoietic cells, can be induced in other cells by proinflammatory factors.^4^ Recent evidence indicates that the immunoproteasome significantly regulates key cellular events, such as inflammatory cytokine secretion,^5^ oxidative stress,^6^ autophagy,^7^ and the metabolism of fatty acids and glucose.^8^

Diabetes and its complications significantly impact public health, affecting approximately 537 million adults aged 20–79, with an additional 541 million experiencing impaired glucose tolerance.^9^ Type I diabetes (T1D) often develops in childhood or adolescence, results from the autoimmune destruction of pancreatic β cells, and affects about 1.1 million young people globally.^10^ Type II diabetes (T2D), the most common type, is caused by the body's cells failing to respond adequately to insulin, namely “insulin resistance”.^11^ It is common in obese middle-aged and elderly patients. Gestational diabetes, characterized by high blood sugar during pregnancy, can occur at any stage and typically resolves after childbirth, but it raises the risk of developing T2D later.^12^ Advancements in medicine and technology offer new hope for diabetes treatment and now focus on managing cardiovascular risks, enhancing insulin resistance, and safeguarding pancreatic β cells, beyond just reducing glucose levels.^13^ However, at present, effective treatments for diabetes are still insufficient, increasing the likelihood of diabetes-related complications.

Emerging evidence indicates that dysregulated immunoproteasome activity plays a key role in diabetes and its complications, offering new avenues for intervention. This review outlines its involvement in disease progression and explores potential therapeutic strategies.

## Overview of immunoproteasome

Around 80% of proteins in eukaryotic cells are degraded by the ubiquitin-proteasome system, containing ubiquitin, ubiquitin activase E1, ubiquitin conjugase E2, ubiquitin-protein ligase E3, 26S proteasome, and ubiquitin dissociation enzyme. 26S proteasome plays a role in protein degradation, and immunoproteasome is a special type of proteasome. Recent studies have found that the immunoproteasome is closely related to tumors, autoimmune diseases, infectious diseases, nervous system diseases, diabetes, *etc*..^5^^,^^14^, ^15^, ^16^, ^17^ Here, we will describe the immunoproteasome from three dimensions: structure, assembly, and function.

### Structure

26S constitutive proteasome is the most common form of proteasome, widely expressed in the cytoplasm and nucleus of eukaryotic cells, and is composed of a 19S RP on each side.^18^^,^^19^ 20S CP has a cylindrical structure, consisting of four heterologous seven-ring α-β-β-α stacks: two outside α rings (α1-α7 subunits) and two inside β rings (β1-β7 subunits). The α-ring of the proteasome forms a gate that controls substrate entry into the proteolytic chamber. It also provides structural stability and serves as a docking platform for regulatory particles, such as the 19S or PA28 complexes. Three subunits of the β ring (β1, β2, and β5) play a catalytic role.^20^

As the catalytic subunits β1, β2, and β5 are replaced by β1i, β2i, and β5i, respectively, immunoproteasome forms.^18^ Compared with constitutive proteasomes, changes in the catalytic subunit could lead to changes in cleavage site preference. Unlike the β1 subunit, which tends to cleave after acidic residues (caspase-like activity), the β1i subunit is more likely to cleave after small hydrophobic residues (branched-chain amino acid–preferring activity). β2 and β2i have similar trypsin-like activity. Though β5 and β5i subunits have chymotryptic-like activity, β5i has a larger specificity pocket for the accommodation of large hydrophobic residues and higher catalytic activity.^21^ Interestingly, these subunits could be mapped in various configurations (usually β1/β2/β5i or β1i/β2/β5i) to form intermediate proteasomes.^22^

The 19S (aka PA700), 11S (aka PA28), and Blm10 (aka PA200) are three main regulatory particles present.^23^ 19S RP can recognize polyubiquitinated substrates and play a gating role on 20S CP, and further degrade proteins in an ATP-dependent manner.^24^^,^^25^ 11S RP in higher eukaryotes contains three subunits named PA28α, PA28β, and PA28γ, which form heteroheptamer PA28αβ and homoheptamer PA28γ respectively.^26^ PA28αβ predominantly associates with the immunoproteasome (iCP), while PA28γ primarily binds to the nuclear constitutive proteasome (cCP), and both facilitate protein degradation in an ATP-independent manner.^27^^,^^28^ Similar to 11S, Blm10 hydrolyzes peptides but not proteins in a non-ATP-dependent way. Besides, it engages in various biological processes, including 20S proteasome assembly, DNA repair, genomic stability, proteasome inhibition, spermatogenesis, and mitochondrial checkpoint regulation.^23^

**Figure 1:**
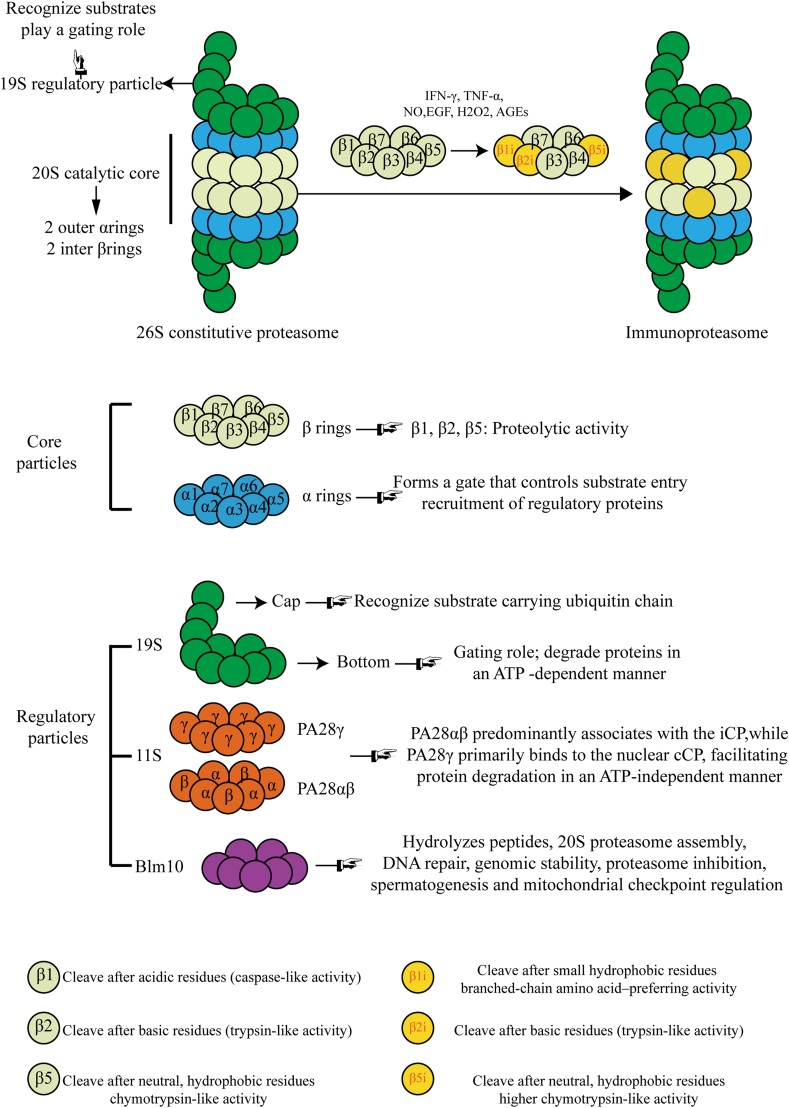
The structure of immunoproteasome. The 26S proteasome consists of a 20S catalytic core (comprising 2 α rings and 2 β rings) and two 19S regulatory particles. Under the stimulation of specific cytokines, subunits β1, β2, and β5 are replaced by β1i, β2i, and β5i, respectively, thereby forming the immunoproteasome. Changes in catalytic subunits lead to alterations in cleavage site preference. AGEs, advanced glycation end-products; NO, nitric oxide; EGF, epidermal growth factor; IFN, interferon; TNF, tumor necrosis factor.

We summarized the above content about the structural features of immunoproteasome as an illustration (Fig. 1).

### Immunoproteasome expression and assembly

**Figure 2:**
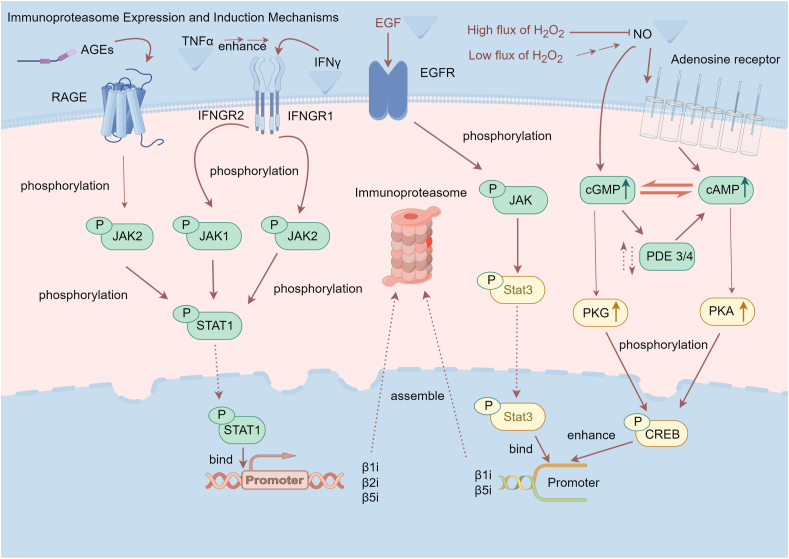
The expression and induction mechanisms of immunoproteasome. IFN-γ, TNF-α, AGEs, EGF, NO, and H_2_O_2_ can directly or assist in inducing the expression of immunoproteasome subunits, thereby increasing the assembly of immunoproteasome. IFN-γ, AGEs, and EGF bind to their respective membrane receptors and then activate the JAK/STAT pathway. The activated STAT binds to the promoter region of the immunoproteasome subunit gene, enhancing its transcription. TNF-α can further enhance this process. NO phosphorylates CREB via the cAMP/PKA and cGMP/PKG pathways. Phosphorylated CREB stimulates the transcription of β1i and β5i. Low flux of H_2_O_2_ enhances this process, while high flux has the opposite effect. AGEs, advanced glycation end-products; NO, nitric oxide; EGF, epidermal growth factor; IFN, interferon; TNF, tumor necrosis factor; RAGE, receptor of advanced glycation end-products; IFNGR, interferon gamma receptor; cAMP, cyclic adenosine monophosphate; cGMP, cyclic guanosine monophosphate; PKA, protein kinase A; PKG, protein kinase G; JAK, Janus kinase; STAT, signal transducer and activator of transcription.

The immunoproteasome is predominantly expressed in hematopoietic cells,^29^ where it plays a crucial role in enhancing the production of major histocompatibility complex I (MHC-I) molecular ligands and facilitating immune surveillance.^30^^,^^31^ In contrast, non-hematopoietic cells can also exhibit immunoproteasome expression, which may be induced by various inflammatory factors. Among these, interferon-gamma (IFN-γ) and tumor necrosis factor-alpha (TNF-α) are the most prevalent inducers, although other factors, like growth factors, nitric oxide, and hydrogen peroxide, have also been implicated in stimulating its expression (Fig. 2).^32^

Inflammation serves as the primary driver of immunoproteasome expression. *In vitro* and *in vivo* studies often utilize IFN-γ to induce immunoproteasome expression.^33^^,^^34^ The inflammatory cytokine IFN-γ activates downstream signaling pathways, including mammalian target of rapamycin (mTOR) and inducible nitric oxide synthase (iNOS), leading to increased protein production. This process generates a significant number of defective ribosome products, which result from errors in translation, folding, and assembly. Immunoproteasome plays a pivotal role in the degradation of defective ribosome products, mitigating the potential harm.^33^ Notably, research indicates that while IFN-β can induce β1i transcription in renal cancer cells, it does not affect β2i transcription. Furthermore, TNF-α has been shown to enhance the expression of immunoproteasome components induced by IFN-γ; however, this effect is not observed when TNF-α is administered in isolation.^35^, ^36^, ^37^

A key signaling pathway involved in the response to various cytokines (including IFN-γ) is the Janus kinase (JAK)–signal transducer and activator of transcription (STAT) pathway. This pathway is integral to processes such as cell proliferation, differentiation, and apoptosis. Upon binding to its receptor, interferon gamma receptor 2 (IFNGR2), IFN-γ phosphorylates JAK kinases, which in turn phosphorylate IFNGR1. This cascade activates transcription factors, notably STAT-1 and interferon regulatory factor 1 (IRF-1). Once activated and translocated to the nucleus, STAT-1 and IRF-1 bind to the promoter regions of the β1i, β2i, and β5i genes, thereby enhancing their transcription and promoting the synthesis of immunoproteasome components.^38^^,^^39^

Epidermal growth factor (EGF), akin to IFN-γ, activates the JAK–STAT signaling pathway upon binding to its receptor, subsequently enhancing the transcription of immunoproteasome subunits β1i and β5i through the activation of transcription factor STAT-3.^38^, ^39^, ^40^ Additionally, nitric oxide plays a crucial role in mitigating oxidative stress-induced apoptosis in endothelial cells by up-regulating immunoproteasome expression. This process involves the activation of protein kinase A (PKA) and protein kinase G (PKG) via cyclic adenosine monophosphate (cAMP) and cyclic guanosine monophosphate (cGMP), leading to the phosphorylation of the transcription factor, cAMP response element-binding protein (CREB), which further stimulates the expression of latent membrane protein 2 (LMP2) and LMP7 encoding immunoproteasome catalytic subunits.^41^

Additionally, advanced glycation end-products (AGEs) have been implicated in the induction of immunoproteasome production in murine macrophages through the JAK–STAT pathway. Investigations utilizing diabetic mouse models have revealed that high glucose perfusion notably elevates the expression of the β5i subunit while concurrently diminishing β1i expression in the free heart. This down-regulation of β1i may be associated with a decrease in nitric oxide (NO) bioavailability, highlighting a complex interplay between metabolic states and immune responses. NO is proven to stimulate the assembly of both proteasome and immunoproteasome. NO/cAMP/PKA and NO/cGMP/PKG pathways phosphorylate CREB and further enhance the transcription of β1i and β5i.^42^^,^^43^ Interestingly, a low flux of H_2_O_2_ facilitates this process, whereas a high flux exerts an inhibitory effect, resulting in a bell-shaped dose–response relationship.^44^

### Function

The immunoproteasome has garnered attention primarily for its crucial role in generating MHC-I antigen peptides. The alteration in cleavage site preferences exhibited by immunoproteasome significantly enhances the production of a diverse library of antigen peptides, thereby bolstering adaptive immunity.^45^^,^^46^ Beyond its function in antigen processing, immunoproteasome is essential in immune function, maintaining cellular protein homeostasis and combating oxidative stress, autophagy, and mitochondrial function.

As for immune function, immunoproteasome modulates the secretion of inflammatory cytokines and the proliferation and differentiation of immune cells. Inhibition or genetic knockout of the β5i subunit in murine models leads to a marked reduction in the secretion of pro-inflammatory cytokines, such as interleukin (IL)-1α/β, IL-6, IL-23, and TNF-α. This inhibition not only impairs the differentiation of Th1 and Th17 cells but also promotes the differentiation of regulatory T cells.^5^^,^^47^ Furthermore, research conducted by Mariella Bockstahler et al in models of autoimmune myocarditis has elucidated the regulatory role of β5i in balancing effector and cluster of differentiation 4-positive (CD4^+^) regulatory T cells within the spleen. This underscores the immunoproteasome's pivotal role in modulating autoimmune responses and influencing disease outcomes.^48^ In addition, the number and viability of B cells in β1i gene knockout mice were reduced, with serious functional defects.^49^ In the state of oxidative stress, the expression and activity of immunoproteasome components increase.^50^ In physiological conditions, proteins are in a low oxidation state. When the balance between pro-oxidation and anti-oxidation systems is broken, excessive reactive oxygen species are generated, followed by the accumulation of a large number of damaged proteins, which can eventually lead to cell death.^51^ For example, in cytokine-induced oxidative stress experiments, β5i gene knockout cells exhibit increased sensitivity to apoptosis. After IFN-γ intervention, a large number of ubiquitinated oxidative damage proteins accumulate in the liver and brain of β5i gene knockout mice, and the central nervous system of β5i gene knockout mice with autoimmune encephalomyelitis is more prone to oxidative damage, suggesting that immunoproteasome plays an important role in regulating protein turnover.^52^^,^^53^ In the cardiac ischemia and reperfusion model mice, β2i KO leads to worse cardiac function through activating parkin–mitofusin 1/2 (Mfn1/2) pathway, which further induces mitochondrial dysfunction, consequently leading to an increase in reactive oxygen species (ROS) and autophagy.^54^ The ubiquitin–proteasome system and the autophagy–lysosome pathway are the two principal proteolytic systems in eukaryotic cells that sustain protein homeostasis. A growing body of evidence suggests that these two systems communicate with each other in the context of various diseases. Recently, a heightened expression of β5i has been linked to increased degradation of phosphatase and tensin homolog (PTEN).^55^ At the same time, there is a higher level of insulin-like growth factor 1 receptor (IGF1R). Both these elements trigger the Akt/mTOR signaling, which has a negative impact on the initiation of autophagy by phosphorylating uncoordinated 51-like kinase 1 (ULK1, S757). Moreover, increased activity of iCP leads to enhanced degradation of autophagy-related protein 5 (ATG5), which further suppresses conversion of cytosolic microtubule-associated protein light chain 3 (LC3) to lipidated LC3 and decreases autophagy.^7^^,^^56^, ^57^, ^58^ We summarize the function of immunoproteasome in Table 1.

**Table 1:** The functions of immunoproteasome.

Function category	Specific function	Mechanism/biological significance	References
Antigen processing and presentation	Protein cleavage to generate peptides	Specifically cleaves intracellular proteins into 8–10 amino acid peptides, ideal for loading onto major histocompatibility complex (MHC) class I molecules for recognition by CD8^+^ T cells	45,46
Immune function	Cytokine regulation	Affects cytokine production (*e.g.*, IL-1α/β, IL-6, IL-23, and TNF-α)	5,47, 48, 49,166
	Proliferation and differentiation of immune cells	Inhibition or deficiency of β5i impairs Th1 and Th17 differentiation while promoting regulatory T cell development; β1i knockout reduces B cell number, viability, and function.	5,47, 48, 49
Proteostasis	Clearance of damaged proteins	More efficiently degrades oxidized or misfolded proteins under stress than standard proteasomes	50,51
	Degradation of regulatory proteins	Promotes turnover of cell cycle and signaling proteins, influencing apoptosis or proliferation	7,55,56
Cellular stress response	Response to oxidative stress	Up-regulated by inflammatory or oxidative conditions; helps restore proteostasis	50,52,53
	Response to heat shock/endoplasmic reticulum stress	Prevents unfolded protein response overload by clearing protein aggregates	52,57
Disease association	Cancer	The immunoproteasome has a dual role in cancer, affecting antigen presentation and immune evasion. It aids in generating MHC class I peptides to boost CD8^+^ T cell anti-tumor responses, but some tumors reduce its subunits to avoid detection. It also helps tumors survive by reducing proteotoxic stress and controlling inflammation.	167, 168, 169, 170, 171
	Heart failure	Immunoproteasome promotes heart failure by degrading ATG5 and PTEN, inhibiting autophagy, and activating hypertrophic signaling. It also enhances renin-angiotensin signaling via ATRAP degradation.	7,118,172
	Neurodegenerative disorders	Immunoproteasome aids neurodegenerative disorders by clearing proteins and managing neuroinflammation. It degrades misfolded proteins such as Aβ and α-synuclein but may exacerbate inflammation and neuronal harm if overactive. Grasping its specific effects could inform new treatments.	173, 174, 175, 176, 177
	Autoimmune diseases	The immunoproteasome contributes to autoimmune diseases by boosting antigen presentation, pro-inflammatory cytokines, and T cell responses. Its increased activity is observed in rheumatoid arthritis, multiple sclerosis, and systemic lupus erythematosus. Targeting its subunits may help restore immune balance and decrease inflammation.	178, 179, 180, 181, 182, 183
	Atherosclerosis	Increased immunoproteasome activity in vascular and immune cells boosts pro-inflammatory cytokine secretion, T cell activation, and foam cell formation.	8,154,184,185

In conclusion, the structure, expression, or functional changes of immunoproteasome are related to numerous phenotypes and diseases. Recent studies have found that the genes encoding immunoproteasome components are related to diabetes susceptibility, and there are also immunoproteasome dysfunctions in diabetic animal models, as we will elaborate on below.

## Role of immunoproteasome in diabetes and its complications

### Immunoproteasome and diabetes

#### Immunoproteasome-related genes and diabetes susceptibility

T1D is an autoimmune disease, with a notable genetic predisposition linked to the human leukocyte antigen (HLA) class II region on chromosome 6. The HLA-DR and HLA-DQ alleles are particularly associated with this condition.^59^^,^^60^ Within this region, the genes for the β1i and β5i proteins play crucial roles in producing MHC-I-related antigen peptides.^61^ However, a randomized controlled trial in a Japanese population found no significant difference in the allele frequency of LMP2 between diabetic patients and healthy controls.^62^ In contrast, a study of 390 Nordic white descendants in the southeastern United States demonstrated a strong association between LMP7 polymorphism and insulin-dependent diabetes, while LMP2 was linked to T1D primarily in individuals with the HLA DR4-DQB1 ∗0302 haplotype, suggesting this connection may not stem from linkage disequilibrium with HLA-DR or HLA-DQ genes.^63^ Further investigation into the Han population in southern China revealed that both LMP2 and LMP7 were linked to T1D independently of the DR3 allele, although this study did not analyze the linkage disequilibrium among other genes within the HLA class II region.^64^^,^^65^ Conversely, other research emphasized the necessity for complete matching of HLA-DR and HLA-DQ alleles between diabetic and control groups, leading to conclusions that LMP2 and LMP7 were not independently associated with T1D.^66^, ^67^, ^68^ The challenge of subgroup matching also raises concerns about sample size adequacy. To address these limitations, a comprehensive cross-analysis involving 11 independent studies was conducted, revealing that LMP2 and LMP7 exhibit weak linkage disequilibrium with DR3/4. Notably, the LMP2-R/H genotype and the LMP7-A/A genotype appeared to correlate more significantly with susceptibility to T1D. Furthermore, the frequency of the LMP7-B/B genotype in the control group was nearly double that in the diabetes group, indicating its potential protective role against the disease.^69^ A meta-analysis encompassing seven studies, with a total of 707 cases and 821 controls, revealed that the dominant model of LMP2 CfoI could potentially serve as a risk factor for T1D among the Asian population. In contrast, the allelic and dominant models of LMP7 G37360T might act as protective elements in the Caucasian population.^70^

Unlike T1D, relatively few studies have been conducted on the relationship between T2D susceptibility and immunoproteasome-encoded genes. Recent studies have shown that functional single-nucleotide polymorphisms of proteasome subunit alpha 6 (PSMA6, which encodes the α6 subunit) increase the risk of T2D in Europeans and Chinese Han nationalities.^71^, ^72^, ^73^ Recently, it has been found that patients with Nakajo-Nishimura syndrome and Japanese autoinflammatory syndrome with lipodystrophy with LMP7 mutations show typical lipodystrophy and relatively increased risk of T2D.^74^^,^^75^

#### Immunoproteasome and apoptosis of islet β cells

T1D results from the apoptosis of islet β cells, driven by a combination of environmental factors and genetic susceptibility.^76^ Substantial evidence highlights viral infection as a significant environmental trigger. During acute viral infections, extensive viral replication and a strong antiviral response can damage the islets, followed by a secondary autoimmune reaction that further destroys β cells. Given the limited regenerative capacity of human islet β cells, this process contributes to diabetes onset.^77^^,^^78^ Viral infections elevate cytokine levels (like IL-1β and IFN-γ/β) in the islet microenvironment of T1D. This increase, possibly due to IFN-1, causes both infected and uninfected β cells to express MHC-I molecules at high levels, present antigenic peptides from β cells, activate CD8^+^ T cells, and trigger autoimmune responses.^79^^,^^80^ Animal studies have demonstrated that IFN-1 can induce diabetes. During the early antiviral response, IFN stimulates the expression of β1i, β2i, and β5i, which are involved in processing MHC-I antigen peptides and may contribute to the development of autoreactive CD8^+^ T cells.^81^^,^^82^ Intervention experiments using IFN-β revealed an increase in intermediate proteasome content in mouse islets and β-cell lines. This activity is regulated by PA700 at high ATP levels and by PA28 at low ATP levels. Additionally, low IL-1β levels enhance intermediate proteasome content. The random combination of catalytic subunits alters cleavage site preferences, potentially leading to the presentation of non-immunogenic autopeptides by MHC-I molecules, which may contribute to the development of T1D. This suggests that intermediate proteasome formation during the early antiviral response is a critical factor in the generation of auto-reactive CD8^+^ T cells.^83^ Additionally, mice with defective immunoproteasome catalytic subunit are more likely to develop T1D after radiation and bone marrow transplantation, and the expression of immunoproteasome can inhibit the occurrence and development of CD8^+^ T cell-mediated autoimmune reaction, suggesting that immunoproteasome seems to play a protective role in T cell-mediated autoimmune reaction.^84^ In conclusion, immunoproteasome dysfunction leads to the apoptosis of islet β cells, resulting in T1D.

Insulin resistance coupled with islet β-cell dysfunction is a hallmark of T2D. Both T1D and T2D exhibit islet inflammation; however, adaptive immunity primarily drives inflammation in T1D, while innate immunity is more significant in T2D.^85^^,^^86^ Chronic inflammation, particularly macrophage-mediated inflammation in T2D, has been confirmed in various animal and human studies.^86^, ^87^, ^88^ Interestingly, the M1 macrophage phenotype contributes to chronic islet inflammation by secreting pro-inflammatory cytokines, such as IL-1β and TNF, whereas the M2 phenotype plays a critical role in islet development and the maintenance of β-cell function.^89^ Coincidentally, recent findings suggest that the immunoproteasome regulates the phenotypic transformation of macrophages. Gene knockout or pharmacological inhibition of β5i enhances M2 macrophage polarization, thereby suppressing pro-inflammatory cytokine secretion and alleviating islet inflammation.^90^^,^^91^ Zinc transporter 8 (ZnT8) is crucial for regulating zinc concentrations in islet β cells and is implicated in β-cell dysfunction and insufficient insulin secretion. Down-regulation of ZnT8 can counteract inflammatory factor-induced β-cell apoptosis, reducing the risk of T2D.^92^ Notably, inflammatory factors induce the expression of the immunoproteasome in β cells, leading to the degradation of ZnT8 and triggering the adaptive unfolded protein response. Inhibition of immunoproteasome activity can prevent the decline in ZnT8 levels under inflammatory conditions and mitigate β-cell apoptosis, indicating that the immunoproteasome may protect β cells by regulating ZnT8 degradation.^92^^,^^93^ Additionally, β5i is found to prevent endoplasmic reticulum stress and apoptosis of islet β cells in T2D by degrading mishandled proinsulin.^94^ In conclusion, the immunoproteasome plays a paradoxical role in T2D, with distinct functions across different cell types, by exacerbating inflammation in macrophages while simultaneously protecting β cells from stress-induced apoptosis. Therapeutic strategies need to finely balance these dual effects to achieve optimal outcomes.

#### Immunoproteasome and glycolipid metabolism

Diabetes is a chronic metabolic disorder marked by elevated blood sugar levels and increased free fatty acids. AMP-activated protein kinase (AMPK) serves as a crucial regulator of intracellular glycolipid metabolism. Numerous studies indicate that AMPK interacts with the ubiquitin-proteasome system to maintain a balance between cellular energy and protein degradation; however, direct and targeted investigations of this relationship are lacking. Recent findings highlight that inhibiting immunoproteasome function can enhance AMPK activation in models of stress-induced cardiac hypertrophy. This activation is linked to improved energy metabolism, suggesting a role for the immunoproteasome in regulating cellular energy processes.^95^, ^96^, ^97^ Obesity, a significant risk factor for insulin resistance and hyperglycemia, has also been studied in this context. LMP7 gene knockout mice demonstrated reduced lipid absorption and fat accumulation following high-fat diets, along with improved glucose tolerance and insulin sensitivity compared with wild-type mice.^91^

Further investigations revealed that the use of ONX0914, a specific inhibitor of β5i, increased the expression of glucose transporter 4 (GLUT4) and insulin receptor substrates in cardiomyocyte membranes while down-regulating CD36 expression. These results imply that LMP7 may be involved in regulating glycolipid utilization by cardiomyocytes under high glucose conditions, and inhibiting LMP7 could enhance their energy metabolism.^98^

#### Immunoproteasome and diabetic complications

A series of pathogenic factors will be produced under the condition of hyperglycemia, leading to the occurrence of diabetic complications. These factors include increased levels of AGEs, oxidative stress, inflammation, endoplasmic reticulum stress, and disrupted protein homeostasis.^99^, ^100^, ^101^ Traditionally, chronic complications are divided into macrovascular complications (cardiovascular and cerebrovascular diseases) and microvascular complications (complications affecting the kidney, retina, and nervous system).^85^^,^^102^

### Diabetic cardiomyopathy

More than half of diabetic patients succumb to cardiovascular disease, with heart failure risks escalating even in the absence of ischemic events and hypertension.^103^^,^^104^ This phenomenon, characterized by myocardial structural and functional alterations independent of hypertension, coronary artery disease, and valvular disease, is termed diabetic cardiomyopathy, first identified in the 1970s.^105^ The primary pathological features of diabetic cardiomyopathy include cardiac hypertrophy and fibrosis, initially presenting as diastolic dysfunction, progressing to systolic dysfunction, and ultimately resulting in heart failure. The development of diabetic cardiomyopathy involves multiple factors: elevated blood glucose levels can lead to increased AGEs, while metabolic disturbances cause lipid accumulation and lipotoxicity in cardiomyocytes. These changes contribute to mitochondrial dysfunction, heightened oxidative stress, and endoplasmic reticulum stress, leading to cardiomyocyte hypertrophy, apoptosis, inflammation, and fibrosis.^105^^,^^106^

Immunoproteasome subunit β1i and β5i are only expressed at low levels in the heart, yet their specific roles remain unclear. High glucose concentrations increase the expression of LMP7 and its chymotrypsin activity, while simultaneously decreasing LMP2 expression, indicating a dysfunction of the immunoproteasome in the hearts of diabetic mice. This imbalance may stem from increased ROS and reduced NO levels in diabetic patients.^43^^,^^107^ Altered energy metabolism is central to diabetic cardiomyopathy, like mitochondrial dysfunction and elevated oxidative stress, which contribute to endothelial dysfunction—a key driver of diabetic cardiomyopathy. This creates a vicious cycle that exacerbates disease progression.^108^, ^109^, ^110^ In diabetic cardiomyocytes, there is a decrease in GLUT4 and an increase in CD36 on the membrane, affecting glucose and lipid uptake. Inhibiting LMP7 reverses this abnormal distribution of transporters under high glucose conditions, enhances energy metabolism, and protects the heart.^98^^,^^111^^,^^112^

Pathological features of diabetic cardiomyopathy include decreased myocardial mass, impaired systolic function, and connective tissue deposition. Myocardial mass loss is primarily driven by chymotrypsin activity; however, studies indicate that an increased β5 subunit does not correlate with high chymotrypsin activity in cardiac tissue.^113^^,^^114^ In streptozotocin-induced diabetic mice, LMP7 expression and associated chymotrypsin activity significantly increase, while MHC protein levels decrease. Inhibiting chymotrypsin activity prevents 75% of MHC protein loss, markedly improving cardiac function without affecting overall blood glucose levels.^43^

Connective tissue deposition leads to diabetic myocardial fibrosis, largely driven by activated myocardial fibroblasts, along with contributions from cardiac, inflammatory, and vascular cells. Factors such as high blood glucose, AGEs, ROS, and renin-angiotensin-aldosterone system activate transforming growth factor-beta (TGF-β) through various mechanisms, promoting fibrosis.^115^ Numerous studies have demonstrated that immunoproteasomes are involved in angiotensin II or high-salt-induced cardiac fibrosis. Inhibiting immunoproteasomes can block the activation of the Akt/TGF-β signaling pathway, thereby reducing fibrosis.^55^^,^^116^, ^117^, ^118^ Moreover, pharmacological inhibition of LMP7 using ONX0914 can inhibit endothelial–mesenchymal transition in T1D diabetic mouse hearts to some extent, at least partly through autophagy activation.^119^

Additionally, the immunoproteasome acts as a bridge between diabetic cardiomyopathy and inflammatory states. Diabetes induces chronic inflammation, where elevated blood glucose and cholesterol levels trigger local inflammation, leading to the recruitment of leukocytes that produce cytokines, chemokines, and ROS.^120^ These factors contribute to disease progression, particularly in diabetic cardiomyopathy, where circulating levels of TNF-α, IL-6, and IL-1β are increased in obese and insulin-resistant patients. The M1 macrophage phenotype, along with Th1 and Th17 cells, also exacerbates the progression of diabetic cardiomyopathy. The immunoproteasome has been implicated in various autoimmune inflammatory diseases, and its inhibition can reduce the levels of proinflammatory cytokines, as observed in some animal models of cardiac remodeling. Moreover, the immunoproteasome regulates the differentiation of Th1 and Th17 cells and the phenotypic conversion of macrophages.^47^^,^^49^^,^^117^^,^^118^^,^^121^ Consequently, it may play a role in the inflammatory progression of diabetic cardiomyopathy, with its inhibition potentially improving the heart's inflammatory states.

The immunoproteasome plays a multifaceted role in diabetic cardiomyopathy, contributing to fibrosis, inflammation, and metabolic dysfunction. Targeting specific subunits like β5i (LMP7) presents a novel therapeutic strategy to alleviate cardiac remodeling and improve outcomes for diabetic patients with cardiomyopathy. Further studies are required to refine these approaches and assess their long-term efficacy and safety.

### Diabetic nephropathy

Diabetic nephropathy is a leading cause of end-stage renal failure, clinically characterized by proteinuria and a progressive decline in glomerular filtration rate, ultimately leading to uremia if untreated.^122^ Elevated blood glucose levels induce inflammation and oxidative stress, affecting various renal cells, including endothelial cells, smooth muscle cells, mesangial cells, podocytes, renal tubular cells, and inflammatory cells, driving the progression of diabetic nephropathy to end-stage renal failure.^123^ This condition is associated with systemic and local inflammation in the kidneys, marked by increased levels of proinflammatory cytokines and chemokines, a common feature across various animal models of diabetic nephropathy.^124^

High blood glucose and AGEs induce immunoproteasome subunit β5i expression, which enhances inhibitor kappa B-alpha (IκBα) degradation and nuclear factor kappa B (NF-κB) activation.^125^^,^^126^ NF-κB, activated through IκBα degradation via ubiquitin-proteasome system, is a key inflammatory factor that regulates the transcription of proinflammatory cytokines, such as IL-18, TNF-α, myeloid differentiation primary response protein 88 (MYD88), and intercellular cell adhesion molecule-1 (ICAM-1). Increased inflammatory mediators and cell infiltration further led to glomerular and tubular injury, interstitial fibrosis, and proteinuria.^127^^,^^128^ ICAM-1 knockout significantly improved the phenotype.^127^

Based on these, the regulatory functions of non-coding RNAs in the LMP7/NF-κB axis have been uncovered, with miR-451 restraining the activity of LMP7/NF-κB^125^ and circ_0000064 inhibiting miR-30c-5p to further activate LMP7/NF-κB, promoting kidney inflammation and fibrosis.^129^

In diabetic nephropathy models, immunoproteasome/NF-κB is also involved in TGF-β/Smad signaling, playing a critical role in fibrotic reactions. Besides, AGEs activate TGF-β through receptor of advanced glycation end-products (RAGE) interaction, promoting renal fibrosis and worsening diabetic nephropathy.^130^^,^^131^ In glomerular mesangial cells, TGF-β activation causes hypertrophy and fibronectin deposition; inhibiting the immunoproteasome reduces TGF-β activation, fibronectin, collagen fibers, and proinflammatory cytokine secretion, thereby curbing fibrosis progression.^125^^,^^132^ Podocyte structural and functional changes are crucial in diabetic nephropathy, with TGF-β inducing epithelial–mesenchymal transition leading to glomerulosclerosis.^133^ Apatinib has been shown to inhibit NF-κB and TGF-β/Smad signaling by down-regulating LMP7, protecting podocytes in streptozotocin-treated mice and high glucose-treated podocytes, and significantly reducing glomerular fibrosis. It also inhibits epithelial–mesenchymal transition in renal tubular cells, improving renal fibrosis and chronic kidney disease progression.^134^^,^^135^

The immunoproteasome, particularly its β5i (LMP7) subunit, plays a central role in the progression of diabetic nephropathy by activating the NF-κB and TGF-β/Smad signaling pathways. These pathways drive inflammation, podocyte injury, epithelial–mesenchymal transition, and fibrosis, ultimately leading to end-stage renal failure. Targeting LMP7 through specific inhibitors like apatinib or leveraging non-coding RNAs such as miR-451 provides promising therapeutic strategies to alleviate kidney injury and slow diabetic nephropathy progression.

### Diabetic retinopathy

Diabetic retinopathy is the most common and serious ocular complication of diabetes and a leading cause of blindness in middle-aged and elderly patients, affecting approximately one-third of individuals with diabetes.^136^, ^137^, ^138^ The progression of diabetic retinopathy is driven by complex pathophysiological changes resulting from high blood glucose levels, including increased AGEs, ROS, renin-angiotensin-aldosterone system activity, inflammatory factors, and vascular endothelial growth factor (VEGF).^136^

Recent studies have shown that the immunoproteasome is expressed in the retina and plays a role in retinal disease progression. In retinal cells and diabetic mouse retinas exposed to high glucose, the expression of the immunoproteasome regulatory component PA28 is increased. Interestingly, immunoproteasome deficiency has a protective effect on the retina following optic nerve injury.^139^, ^140^, ^141^ In an angiotensin II-induced retinopathy mouse model, knockout of β2i or β5i improved retinal vascular permeability, inflammation, and oxidative stress.^142^ Key factors such as PTEN, NF-κB, and TGF-β are involved in angiotensin II-induced retinopathy. The knockout of immunoproteasome subunits reduces the degradation of PTEN and IκBα, blocking NF-κB activation.^143^^,^^144^ The NF-κB signaling pathway is a crucial regulator of IL-6, NADPH oxidase, and VEGF, which modulate inflammation, oxidative stress, and vascular function. Therefore, the inhibition of the immunoproteasome may potentially improve the progression of diabetic retinopathy.^145^^,^^146^

**Figure 3:**
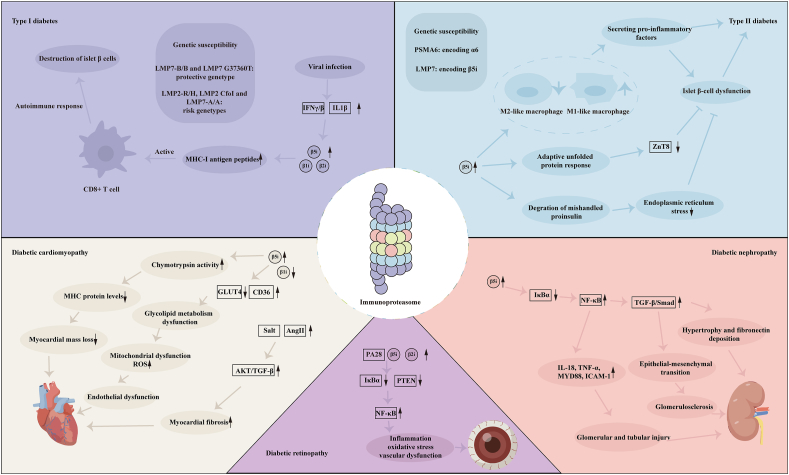
The role of immunoproteasome in diabetes and its complications. Regarding type I diabetes, the immunoproteasome activates CD8^+^ T cells, leading to the apoptosis of islet β cells. As for type II diabetes, immunoproteasome exhibits a dual role, promoting the secretion of pro-inflammatory cytokines and protecting islet β cells from apoptosis. Additionally, the coding genes of immunoproteasome subunits are associated with the onset of diabetes. Changes in IκBα/NF-κB, TGF-β/Smad, and glycolipid membrane receptors play a crucial role in the pathogenesis of diabetic complications. IFN, interferon; MHC-I, major histocompatibility complex I; TGF, transforming growth factor; ROS, reactive oxygen species.

The section about the role of immunoproteasome in diabetes and its complications was summarized in Figure 3.

## Potential therapeutic value

At present, several immunoproteasome inhibitors have been discovered and developed, including short peptide covalent binding inhibitors, short peptide non-covalent binding inhibitors, and non-peptide inhibitors.^147^^,^^148^ The short peptide covalent binding inhibitors are the most common immunoproteasome inhibitors at present, which are divided into epoxyketone peptides and other peptide-based compounds. Some non-specific drugs have also been found to inhibit the subunit production of immunoproteasome, including apelin and resveratrol (3,4’,5-trihydroxy-stilbene, RES).^134^^,^^149^ Recent studies have found that immunoproteasome inhibitors play a therapeutic role in inflammatory and autoimmune diseases (including rheumatoid arthritis, inflammatory bowel disease, and systemic lupus erythematosus^147^^,^^150^^,^^151^), cardiovascular diseases (including heart failure,^55^ hypertension,^152^ myocarditis,^153^ and atherosclerosis^154^), and diabetes along with its complications.^119^ Based on our thorough search on the PubMed database, we summarize three promising drugs in diabetes as shown in Table 2.

**Table 2:** Immunoproteasome inhibitors in the treatment of diabetes and its complications.

Inhibitor	Chemical formula	Target	Effective against	IC50	Reference
PR-957/ONX0914	C_31_H_40_N_4_O_7_	β5i	Diabetic cardiomyopathy	5.7 nM	119
Apelin-13	C_69_H_111_N_23_O_16_S · C_2_F_3_O_2_	β5i	Diabetic nephropathy	0.37 nM	134,158
Resveratrol	C_14_H_12_O_3_	β1i, β2i, β5i	Type I diabetes, type II diabetes, diabetic nephropathy, diabetic neuropathy, diabetic retinopathy, diabetic liver damage	NA	149

Note: IC50, half maximal inhibitory concentration.

### PR-957/ONX0914

ONX0914 is the first epoxyketone peptide β5i selective immunoproteasome inhibitor.^155^ It can inhibit the phosphorylation of extracellular signal-regulated kinase 1/2 (ERK1/2) and P38, thereby inhibiting STAT1/3 activation and reducing the secretion of collagen fibers.^156^ ONX0914 can also regulate TGF-β/Smad signaling pathway to inhibit various fibrotic reactions. Regarding glycolipid metabolism, ONX-0914 decreases intestinal triglyceride absorption and gastric emptying, thereby leading to a reduction in white adipose tissue quantity. Along with the decrease in white adipose tissue mass, enhancements in metabolic syndrome markers are noted, such as reduced levels of plasma triglycerides, insulin, and fasting blood glucose.^8^ Our recent study has demonstrated that ONX0914 ameliorates diabetic cardiomyopathy by restraining endothelial–mesenchymal transition.^119^ In summary, ONX0914 has a protective effect on glycolipid metabolism and cardiac function in mouse models. More clinical investigations need to be done to explore the therapeutic value in corresponding clinical patients.

### Apelin-13

Apelin, an adipokine, holds a crucial role in lipid metabolism.^157^ Apelin-13, a subtype of apelin, functions in regulating blood glucose level.^158^ Initially, it was found that apelin-13 can decrease blood glucose levels and alleviate insulin resistance.^159^ Yin et al first investigated the role of apelin-13 in immunoproteasome and diabetic nephropathy. They demonstrated that apelin-13 exerted a therapeutic effect on diabetic nephropathy through inhibiting epithelial–mesenchymal transition of podocytes by suppressing β5i and further down-regulating the expression and activation of TGFβ/Smad.^134^ Additionally, Gao et al indicated that apelin-13 eased diabetic nephropathy by boosting the production of NO and reducing the fibrosis of kidney tissues.^158^ Similar to ONX0914, more clinical studies are needed to confirm the real therapeutic efficacy of apelin-13 on diabetic nephropathy.

### RES

RES, a naturally occurring phytoalexin, can be predominantly extracted from cereals, fruits, vegetables, dry legumes, and plant-derived beverages like tea, coffee, and wine.^149^ RES displays a broad spectrum of pharmacological effects, including anti-cancer, anti-cardiovascular disease, and anti-diabetes activities.^160^, ^161^, ^162^ Regarding diabetes, RES has been found to improve insulin resistance,^163^ enhance glucose and lipid metabolism,^164^ and protect islet β cells from apoptosis.^165^ Based on these, RES has been widely applied in the treatment of diabetes and its complications. Interestingly, although extensive basic research has been done to explore the molecular mechanism of RES on diabetes, the inhibitory role of RES on immunoproteasome was not reported until 2019. Chen et al show that RES can inhibit the expression of β1i, β2i, and β5i, and suppress the activity of immunoproteasome,^116^ which may supplement its therapeutic mechanism in diabetes. Although there is no direct evidence that RES improves diabetes through inhibiting immunoproteasome, we speculate that the therapeutic effect of RES on diabetes is produced by inhibiting the immunoproteasome pathway based on the above research results.

## Conclusions and prospects

This article comprehensively reviews the relationship between immunoproteasome and diabetes, primarily from genetic susceptibility, glycolipid metabolism dysfunction, islet β cell apoptosis, diabetic complications, and potential therapeutic inhibitors. Inhibition of immunoproteasome function not only has the potential to improve the energy metabolism of diabetic cells but also inhibits the secretion of proinflammatory cytokines, reduces immune cell infiltration, and prevents fibrosis changes in target organs. ONX0914, apelin-13, and RES exhibit promising prospects for clinical application in diabetes and its complications. More studies are worthy of being conducted to confirm the actual effect or develop novel inhibitors for diabetic treatment.

However, many questions still need to be solved: i) whether the production of intermediate proteasome will lead to the presentation of islet β cell autoantigens should be clarified; ii) the role of RES in diabetes and immunoproteasome remains to be determined; iii) immunoproteasome can degrade oxidative damage proteins, and whether inhibiting immunoproteasome will aggravate oxidative stress damage, or can reduce ROS production in the upstream (such as improving metabolism) requires investigation; iv) the mechanism of immunoproteasome dysfunction in diabetic heart, and the effect and mechanism of inhibiting immunoproteasome activity on diabetic cardiomyopathy are gaining attention; v) the role of immunoproteasome in other diabetic complications also awaits investigation. With the deepening of research on immunoproteasome and diabetes, the role of immunoproteasome in the occurrence and development of diabetes will be discussed more comprehensively in the future, so as to provide new therapeutic targets and strategies for diabetes and reduce the damage of diabetes to the population.

## CRediT authorship contribution statement

**Mengwen Wang:** Methodology, Writing – review & editing. **Lingyun Luo:** Writing – original draft, Investigation, Visualization. **Lei Dai:** Funding acquisition, Investigation, Conceptualization. **Hesong Zeng:** Resources, Conceptualization, Supervision, Methodology. **Hongjie Wang:** Writing – review & editing, Conceptualization, Supervision.

## Funding

The work was supported by the 10.13039/501100001809National Natural Science Foundation of China (No. 82270368), Hubei Provincial Natural Science Foundation of China (No. 2024AFB062), and the Cultivation Fund by Tongji Hospital affiliated to Tongji Medical College HUST (Hubei, China) (No. 2023B04).

## Conflict of interests

The authors declared no relevant conflict of interests.
